# Lupeol Counteracts the Proinflammatory Signalling Triggered in Macrophages by 7-Keto-Cholesterol: New Perspectives in the Therapy of Atherosclerosis

**DOI:** 10.1155/2020/1232816

**Published:** 2020-09-27

**Authors:** Sarmistha Saha, Elisabetta Profumo, Anna Rita Togna, Rachele Riganò, Luciano Saso, Brigitta Buttari

**Affiliations:** ^1^Department of Cardiovascular and Endocrine-Metabolic Diseases, and Aging, Italian National Institute of Health, Rome 00161, Italy; ^2^Department of Physiology and Pharmacology “Vittorio Erspamer”, Sapienza University of Rome, Rome 00161, Italy

## Abstract

Macrophage activation and polarization play a central role in atherosclerotic plaque fate. The M1/M2 activation phenotypes represent two profiles of the macrophage polarization state. During atherosclerosis regression or stabilization, macrophages switch from M1 proinflammatory phenotype to M2 anti-inflammatory reparative one. Here, we investigated whether the natural compound lupeol, a pentacyclic triterpene, induces phenotypical and functional changes in human M1 macrophages and counteracts the proinflammatory signalling triggered by 7-keto-cholesterol (7KC), a major product of oxidative stress-mediated cholesterol oxidation. Flow cytometric and immunochemical analysis showed that the treatment with lupeol of M1 monocyte-derived macrophages *M*_(IFN-*γ*/LPS)_ specifically stimulated these cells to upregulate the expression of the anti-inflammatory cytokines interleukin- (IL-)10 and TGF-*β*, and of the scavenger receptor CD36, whereas downregulated the proinflammatory cytokine IL-12 and the M1 activation marker HLA-DR. Pretreatment of macrophages with lupeol prevented the release of IL-12, IL-1*β*, and the upregulation of HLA-DR expression triggered by 7KC and increased the IL-10 production and CD36 expression. This treatment also prevented the impairment of endocytosis triggered by 7KC and prevented 7KC-induced foam cell formation by reducing the lipid droplet accumulation in M1-polarized THP-1 macrophages, whereas showed an additive effect in reactive oxygen species (ROS) production. Western blotting analysis of autophagy markers LC3-I/II and p62-SQSTM1 in M1-polarized THP-1 macrophages demonstrated that lupeol activated autophagy as indicated by increased LC3-II levels, and by marked inhibition of p62. These findings indicate that lupeol has a cytoprotective effect on 7KC-proinflammatory signalling by efficiently switching the macrophage polarization toward an anti-inflammatory phenotype, probably through the activation of the autophagy pathway by increasing ROS production, the reduction of cellular lipid accumulation, and an overall reduction of proinflammatory phenotype. Thus, our data demonstrating an anti-inflammatory and immunomodulatory activity of lupeol in human M1 macrophages suggest its usefulness as an adjunctive drug in the therapy of atherosclerosis.

## 1. Introduction

Atherosclerosis is a chronic inflammatory disease characterized by the accumulation of immune cells such as macrophages and foam cells in the intima of the vessel wall [1, 2]. Both cells contribute to the classical atherosclerotic plaque destabilization and rupture by secreting proinflammatory cytokines and matrix metalloproteinases. The fate of atherosclerotic plaques is highly dependent on the balance between the recruitment and activation of monocyte-derived macrophages and their clearance from the vessel wall [3, 4]. A characteristic feature of macrophages is their plasticity due to the ability to reversibly change their phenotype and function in response to signals in the microenvironment. The so-called M1 and M2 activation phenotypes represent two profiles of the macrophage polarization state [5]. The predominant production of proinflammatory cytokines and reactive oxygen species (ROS) by the M1 macrophage phenotype promotes atheroma formation, while the expression of immunosuppressive cytokines and growth factors by the M2 state resolves atheroma by stimulating angiogenesis and phagocytosis [4, 6, 7]. Thus, the M1/M2 phenotype balance is possibly responsible for cholesterol crystal formation or disappearance [8].

The molecular and cellular mechanisms involving macrophage polarization and activation play a central role in plaque progression and stability. Recent studies have shown that oxysterols, oxidative stress-mediated cholesterol oxidation products, which are abundant in atherosclerotic lesions, may switch macrophage phenotype towards a proinflammatory profile [9]. We recently demonstrated that 7-keto-cholesterol (7KC), the most abundant cholesterol oxidation product within atherosclerotic plaques [10, 11], is responsible for switching the macrophage phenotype towards a proinflammatory profile [9].

Few natural compounds such as apigenin, curcumin, and resveratrol have been shown to inhibit the proinflammatory functions of adipose tissue macrophages which were polarized to M1 cells by lipopolysaccharide [12–16]. A range of synthetic chemical entities and natural plant-derived compounds have been characterized for their ability to modulate inflammation and related signalling [12]. Moreover, preclinical as well as clinical studies have shown that the dietary phytochemicals lower the risk of developing coronary heart diseases [12]. Hence, this evidence prompted researchers to investigate the potential therapeutic interventions with plant-derived natural products.

Although their ability to modulate the M1/M2 phenotype is clear, it is still unclear whether these molecules could be practically effective as therapeutic agents for the treatment of atherosclerosis-related inflammation and the exact mechanisms behind their action. Therefore, it is worthwhile to add mechanistic insights into the effects of natural plant-derived compounds to modulate macrophage polarization.

In this regard, lupeol, a pentacyclic triterpene—widely available in fruits such as mango, red grapes, olives, and strawberry as well as in vegetables such as cucumber, white cabbage, and tomato—has been shown to exhibit potent anti-inflammatory activity by decreasing the release of proinflammatory cytokines such as TNF-*α* and IL-*β* in lipopolysaccharide-(LPS-)treated macrophages in rat and mouse models of inflammation [17–19]. Lupeol has also been shown to inhibit latent membrane protein 1-induced NF-*κ*B activation and consequently to switch proinflammatory macrophages into anti-inflammatory phenotype in experimental inflammatory bowel disease [20]. Furthermore, it has been also suggested that a derivative molecule of lupeol induces cell death in a cancer cell line by inducing autophagy rather than apoptosis and necrosis [21]. Growing evidence demonstrates that dysfunctional autophagy plays an important role in atherosclerotic plaque destabilization and the overall development of the disease [22–26]. Recently, it has also been revealed that the induction of autophagy in macrophages may have a plaque-stabilizing effect [24]. Therefore, the activation of the autophagy pathway could be a potential therapeutic strategy for atherosclerosis. This motivated us to investigate whether lupeol modulates the phenotype and function of human M1 macrophages by counteracting the 7KC-proinflammatory signalling. In particular, we investigated whether lupeol is able to induce autophagy in 7-KC-treated M1 macrophages.

In this work, we used immunochemical and flow cytometric analysis to investigate the endocytosis, ROS, cytokine production, surface marker expression, cellular lipid levels, and autophagy markers in human classically activated macrophages (*M*_(IFN-*γ*/LPS)_), also known as M1 macrophages pretreated with lupeol and exposed to the proinflammatory stimulus 7KC.

## 2. Materials and Methods

### 2.1. Reagents

Recombinant human (rh) macrophage colony-stimulating factor (M-CSF) was purchased from R&D System (Minneapolis, MN). Fetal bovine serum (FBS) was purchased from Hyclone Laboratories (Logan, UT). Anti-CD14-coated microbeads, human IFN-*γ*1b (IFN-*γ*), fluorescein isothiocyanate- (FITC-) conjugated mAbs to human leukocyte antigen-D region related (HLA-DR), and VioGreen-conjugated mAbs to CD36 were purchased from Miltenyi Biotec (Gladbach, Germany). Lupeol, 7KC, phorbol 12-myristate 13-acetate (PMA), lipopolysaccharides from Escherichia coli (LPS), FITC-dextran, and 2′,7′-Dichlorofluorescein Diacetate (H2DCF-DA) were purchased from Sigma-Aldrich (Milan, Italy). Sytox Blue nucleic acid stain and 4′,6-diamidino-2-phenylindole (DAPI) were purchased from Thermo Fisher Scientific (Waltham, Massachusetts, USA).

### 2.2. Preparation of Human *M*_(IFN-*γ*/LPS)_ Macrophages

Peripheral blood mononuclear cells (PBMCs) were obtained from buffy coats of healthy blood donors collected from the Transfusion Center at the Sapienza University of Rome [27]. The study was conducted in accordance with the Helsinki Declaration of 1975 and 1983. Briefly, PBMCs were isolated by density gradient and CD14^+^ monocytes were purified by incubating PBMCs with anti-CD14-coated microbeads, followed by sorting with a magnetic device. Monocytes were then induced to differentiate in the presence of rhM-CSF to obtain monocyte-derived macrophages. Human leukemic cell line THP-1 (ATCC, Manassas, VA, USA) was grown in a complete medium (RPMI 1640 supplemented with 1% nonessential amino acids, sodium pyruvate (1%), Penicillin (50 units/mL), Streptomycin (50 *μ*g/mL), 2-mercaptoethanol (5 × 10^−5^ M) and 10% FBS) and prior to the experiments; THP-1 cells were differentiated to macrophages by incubating with 10 ng/mL PMA in culture medium for 48 hours, followed by a wash with phosphate-buffered saline (PBS) and finally grown in culture medium for 24 hours. Human monocyte-derived macrophages (primary macrophages) or THP-1 macrophages were then polarized towards the M1 phenotype using 10 ng/mL IFN-*γ* and 10 ng/mL toll-like receptor 4 ligand LPS M_(IFN-*γ*/LPS)_ for an additional 24 h. All cells were cultured in complete medium, washed with warm PBS, and harvested using TrypLE™ Express Enzyme (Gibco, Grand Island, NY, USA).

### 2.3. Treatment of *M*_(IFN-*γ*/LPS)_ Macrophages with Lupeol and/or 7-Keto-Cholesterol

Human primary *M*_(IFN-*γ*/LPS)_ macrophages and THP-1 *M*_(IFN-*γ*/LPS)_ macrophages were treated or not with lupeol (10-50 *μ*M) for 1 hour at 37°C and 5% CO_2_. The cells were then stimulated with 7KC dissolved in ethanol (15 *μ*M) for 20 hours. The inflammatory stimuli LPS (200 ng/mL) and ethanol were used as controls. Cell viability was measured employing the Trypan blue exclusion assay, and cell morphology was checked by a light microscope.

### 2.4. Secretome Profile of Cytokines in Macrophage Culture Supernatants

Supernatants from human primary *M*_(IFN-*γ*/LPS)_ macrophages (7 × 10^5^ cells per mL) pretreated with lupeol for 1 hour and then exposed to 7KC (20 nm/L) for a further 20 hours in 24-well plates were collected, centrifuged, and stored at -80°C. The levels of IL-12 p70, IL-1*β*, IL-10, and TGF-*β* into the conditioned medium were determined by enzyme-linked immunosorbent assay (ELISA; OptEIA kits; BD Biosciences) following the manufacturer's instructions. The limits of detection were as follows: IL-10 and IL-1*β*: 16 pg/mL; IL-12p70: 7.8 pg/mL; and TGF-*β*: 62.5 pg/mL.

### 2.5. Flow Cytometric Analysis of Macrophage Phenotype

To determine macrophage phenotypic surface markers, human primary *M*_(IFN-*γ*/LPS)_ macrophages were stained with anti-HLA-DR and anti-CD36 mAbs or with isotype-matched control mAbs for 30 minutes at 4°C and then analyzed by flow cytometry on a Gallios Flow Cytometer (Beckman Coulter) equipped with three lasers (488 nm, 638 nm, and 405 nm), and the results were further analyzed by the use of fluorescence-activated cell sorting (FACS) Kaluza analysis software (Beckman Coulter).

### 2.6. Flow Cytometric Analysis of Macrophage Endocytosis

Macrophage mannose receptor-mediated endocytosis was determined by the method as previously described [28]. In brief, human primary *M*_(IFN-*γ*/LPS)_ macrophages (1 × 10^6^ cells/mL) were incubated with FITC-dextran (1 mg/mL) for 45 min at 37°C. Internalization ability was analyzed as the percentage and the mean fluorescence intensity (MFI) of FITC-positive cells by flow cytometry and then analyzed by flow cytometry on a Gallios Flow Cytometer (Beckman Coulter). The results were further analyzed by the use of fluorescence-activated cell sorting (FACS) Kaluza analysis software (Beckman Coulter). Dead cells were excluded by 1 *μ*M Sytox Blue nucleic acid staining.

### 2.7. Flow Cytometric Analysis of Intracellular Lipid Levels

*In vitro* model of foam cell formation induced by the oxysterol mixture 7*β*-hydroxycholesterol and 7KC was previously described by Yuan et al. [29]. Here, THP-1 *M*_(IFN-*γ*/LPS)_ macrophages were treated with only 7KC. In brief, THP-1 *M*_(IFN-*γ*/LPS)_ macrophages (1 × 10^6^ cells/mL) were pretreated with lupeol for 1 hour and then exposed to 7KC (20 nm/L) for a further 20 hours in complete medium. Cells were stained with LipidSpot™ 488 Lipid Droplet Stains according to the manufacturer's instructions (Biotium, USA). LipidSpot™ dyes are fluorogenic neutral lipid stains that rapidly accumulate in lipid droplets, where they become brightly fluorescent (Abs/Em: 427/585 nm). After 30 min of incubation in the dark at 37°C, cells were centrifuged and the pellet was washed twice with ice-cold PBS/FCS and stained with DAPI (4 *μ*g/mL) to exclude dead cells. At least 5 × 10^3^ cells/sample was analyzed by flow cytometry (Gallios Flow Cytometer; Beckman Coulter).

### 2.8. Flow Cytometric Analysis of Reactive Oxygen Species (ROS) Production

The production of ROS in human primary *M*_(IFN-*γ*/LPS)_ macrophages was measured through H2DCF-DA staining. In brief, macrophages (1 × 10^6^ cells/mL) were incubated with H2DCF-DA at a final concentration of 2.5 *μ*M. After 45 min of incubation in the dark at 37°C, cells were centrifuged, and the pellet was washed twice with ice-cold PBS/FCS, and then fixed with 1% formaldehyde. At least 5 × 10^3^ cells/sample were analyzed by flow cytometry (Gallios Flow Cytometer; Beckman Coulter). DCFDA fluorescence intensity was measured in FL-1 with an excitation wavelength of 488 nm and an emission wavelength of 530 nm.

### 2.9. Western Blot Analysis of Macrophage Lysates for Autophagy Markers

*M*_(IFN-*γ*/LPS)_ polarized THP-1 macrophages and primary macrophages were lysed on ice in CelLytic buffer (Sigma Aldrich) plus protease and phosphatase inhibitors (protease inhibitor cocktail: 1 mM sodium fluoride, 1 mM sodium orthovanadate, and 1 mM sodium molybdate; 1 mM phenylmethylsulfonyl fluoride; and 1 mM phosphoinositidase C (Sigma Aldrich)). Other drugs used were chloroquine (ChQ; 50 *μ*M), 3-metyladenine (3-MA; 5 mM), and rapamycin (Rapa; 2 *μ*M), all purchased from Selleckchem (Verona, Italy). Lysates were incubated for 20 min at 4°C and centrifuged for 15 min at 16,000 × g and 4°C to pellet the insoluble material. Samples were then stored at −20°C until use. At the time of analysis, the samples were denatured in 4 × Laemmli Sample Buffer (Bio-Rad), added with 50 mM DTT, and then heated for 5 min at 95°C. Samples were then loaded per lane in equal volumes and separated by electrophoresis in 4-15% Mini-PROTEAN® TGX Stain-Free™ Precast Gels (Bio-Rad, Milan, Italy). Protein samples were then transferred electrophoretically in Towbin buffer (25 mM Tris, 192 mM glycine, pH 8.3, and 20% (*v*/*v*) methanol) to polyvinylidenedifluoride membranes (Millipore, Milan, Italy). After protein transfer, membranes were imaged for stain-free staining and total protein was quantified using Imagelab 6.0.1 (Bio-Rad) to correct for possible protein loading inaccuracy. The membranes were then blocked with 2% (wt/vol) low-fat milk in Tris-buffered saline (137 mM NaCl, 20 mM Tris·HCl, pH 7.6) containing 0.1% Tween 20 (TBS-T) for 1 h at room temperature. The membranes were further incubated overnight at 4°C with the primary antibodies rabbit anti-LC3B (at dilution 1 : 2000) and mouse anti-p62-SQSTM1 (1 : 1000). All the antibodies were procured from Novus Biologicals (Bio-Techne Ltd, Milan, Italy). After three washes with TBS-T, the membranes were incubated for 2 h, at room temperature, with an alkaline phosphatase-linked secondary antibody, specific to rabbit and mouse IgG (1 : 10000). Protein immunoreactive bands were visualized by chemifluorescence with the Clarity Western ECL Substrate (Bio-Rad) in a ChemiDoc Imaging System (Bio-Rad). Some membranes were reprobed with a monoclonal anti-*β*-actin Ab (1 : 5000; Sigma) for equal protein loading control. The optical density of the bands was quantified with the Imagelab 6.0.1 (Bio-Rad). The results were normalized to total protein and expressed as the relative amount compared with control.

### 2.10. Statistical Analysis

Mean values and standard deviations (SD) were calculated for each variable under study. All the statistical analysis was performed by GraphPad Prism 8 software (San Diego, CA, USA). Normally distributed data were analyzed using one-way ANOVA with a Tukey post hoc test. Values of *P* < 0.05 were considered statistically significant.

## 3. Results

### 3.1. Lupeol Skews *M*_(IFN/LPS)_ towards Anti-Inflammatory Phenotype and Counteracts the Proinflammatory Signalling Triggered in Macrophages by 7-Keto-Cholesterol

**Figure 1:**
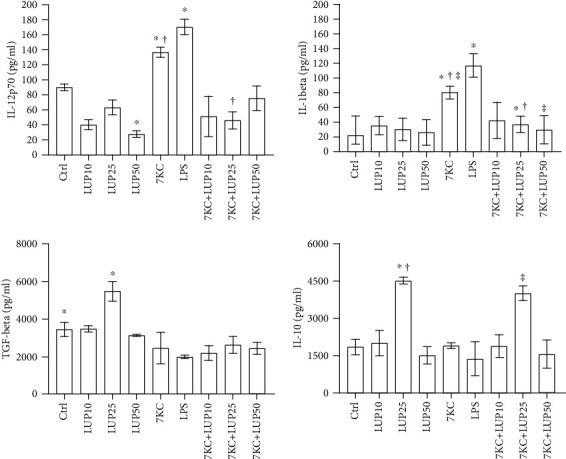
Cytokine production in *M*_(IFN-*γ*/LPS)_ pretreated or not with lupeol before stimulation with 7-keto-cholesterol. Primary *M*_(IFN-*γ*/LPS)_ macrophages (7 × 10^5^ cells per mL) were stimulated or not with lupeol at the different concentrations for 1 hour and further stimulated with 7KC in complete medium. Supernatants were collected after 20 hours to measure cytokines by specific ELISA experiments. Results are expressed as mean value ± SD of 3 independent experiments. *P* values were tested by one-way ANOVA. ^∗^^†^ lupeol 10 *μ*M vs. lupeol 25 *μ*M; ^‡^ 7KC-treated group vs. 7KC+lupeol 25 *μ*M.

Lupeol is a pentacyclic triterpene with potent anti-inflammatory activity [16]. Pentacyclic triterpenes have been found to exhibit anti-inflammatory activity although their role in macrophage polarization and the mechanism by which this process could take place has yet to be elucidated [20]. In order to confirm the hypothesis that lupeol could be able to exhibit antiatherosclerotic activity by inhibiting inflammatory changes, human CD14^+^ monocytes were differentiated into macrophages, polarized toward M1-like phenotype (primary *M*_(IFN-*γ*/LPS)_ macrophages), and further treated with different concentrations of lupeol. Dose-response experiments demonstrated that 50 *μ*M was the highest tolerated concentration of lupeol that did not affect macrophage viability in the Trypan blue exclusion assay and or cell morphology (see Figure S1 in the Supplementary Material for comprehensive result analysis). Therefore, we selected three different concentrations of 10, 25, and 50 *μ*M of lupeol to investigate the total macrophage secretory capacity by determining the secretome profile of cytokines in the cell supernatants by ELISA. After incubation of the macrophages with 7KC or LPS, we observed a significant increase in the release of the proinflammatory cytokines IL-12, IL-1*β*, whereas the release of the anti-inflammatory cytokines TGF-*β* and IL-10 was reduced as compared with unstimulated *M*_(IFN-*γ*/LPS)_ cells (in Figure 1). Of note, the treatment of *M*_(IFN-*γ*/LPS)_ macrophages with lupeol at 25 *μ*M induced a significant increase of IL-10 and TGF-*β* production, whereas at 50 *μ*M significantly decreased IL-12 (in Figure 1). The pretreatment with lupeol prevented the increase of IL-12 (at 25 *μ*M) and IL-1*β* (at 25 and 50 *μ*M) in the cell supernatants induced by 7KC-treated macrophages. At 25 *μ*M lupeol increased significantly the secretion of IL-10 in 7KC-treated macrophages (in Figure 1).

**Figure 2:**
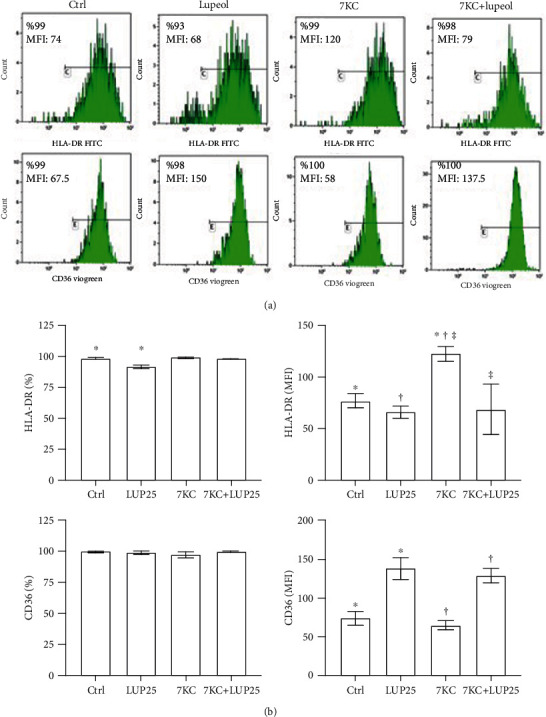
Flow cytometric analysis of surface marker expressions on *M*_(IFN-*γ*/LPS)_ macrophages. Lupeol skews primary *M*_(IFN-*γ*/LPS)_ macrophage phenotype towards an anti-inflammatory phenotype and prevents 7-keto-cholesterol (7KC) induced changes in *M*_(IFN-*γ*/LPS)_ macrophages. *M*_(IFN-*γ*/LPS)_ primary macrophages were stimulated or not with lupeol at 25 *μ*M for 1 hour and further stimulated with 7KC and then analyzed for HLA-DR and CD36 expressions by flow cytometry. (a) The results of one representative experiment of three are shown. The number in the histograms shows the percentages of positive cells (%) and the mean fluorescence intensity (MFI). (b) Flow cytometric analysis of surface marker expression on *M*_(IFN-*γ*/LPS)_ macrophages. Results are expressed as % and MFI (mean ± SD; *n* = 3). *P* values were calculated by one-way ANOVA with a Tukey post hoc test.

The flow cytometric analysis of the M1- or M2-related surface antigens HLA-DR and CD36 was conducted in primary *M*_(IFN-*γ*/LPS)_ macrophages (in Figure 2). Analysis of surface antigen expressions of macrophages shows a reduction in the percentage of HLA-DR positive cells (*P* < 0.024) and an increase in the CD36 expression (MFI) (*P* < 0.048) after the treatment with 25 *μ*M lupeol. As expected, 7KC induced an increase in the HLA-DR expression (MFI) (*P* < 0.009), but it did not alter the expression of the CD36. The pretreatment with 25 *μ*M lupeol prevented the increase of HLA-DR expression by 7KC (*P* < 0.034) and simultaneously increased the expression of CD36 (*P* < 0.04) on *M*_(IFN-*γ*/LPS)_ macrophages. These results confirm that lupeol exerts anti-inflammatory activity by switching *M*_(IFN-*γ*/LPS)_ macrophage phenotype toward an anti-inflammatory phenotype.

### 3.2. Lupeol Prevents the Impairment of Endocytosis in 7-Keto-Cholesterol-Treated *M*_(IFN-*γ*/LPS)_ Macrophages

**Figure 3:**
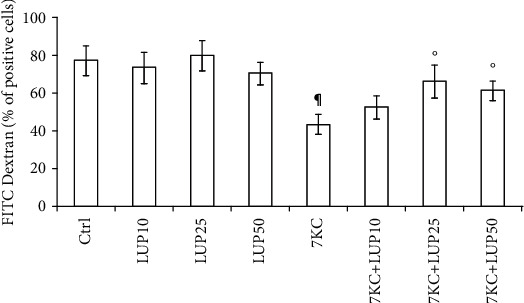
Analysis of *M*_(IFN-*γ*/LPS)_ macrophage endocytosis. Lupeol prevents the impairment of endocytosis induced by 7-keto-cholesterol (7KC) in primary *M*_(IFN-*γ*/LPS)_ macrophages. Human primary *M*_(IFN-*γ*/LPS)_ macrophages were incubated with lupeol (10, 25, and 50 *μ*M) followed by stimulation with 7KC (15 *μ*M) and then added with FITC-dextran. The cellular uptake was then analyzed by flow cytometry. Results are expressed as a percentage of positive cells (%) and mean fluorescence intensity (MFI) (mean ± SD; *n* = 3). *P* values were tested by one-way ANOVA. ^∗^Untreated vs. lupeol; ^¶^Untreated control vs. 7KC-treated group; °7KC-treated group vs. 7KC+lupeol treated groups.

Endocytosis is a crucial factor in macrophage-mediated host defence, which involves the internalization and destruction of pathogens. Unlike anti-inflammatory, proinflammatory macrophages have shown less endocytic ability [27]. Flow cytometric analysis showed that the unstimulated primary *M*_(IFN-*γ*/LPS)_ macrophages largely resulted positive for the FITC-dextran uptake. As expected, we found a significant decrease in the uptake of FITC-dextran due to the stimulation of *M*_(IFN-*γ*/LPS)_ macrophages with the proinflammatory signalling triggered by 7KC (in Figure 3). Of note, the pretreatment of the cells with lupeol was able to prevent the reduction of endocytosis ability induced by 7KC, thus suggesting a less proinflammatory state of macrophages (in Figure 3).

### 3.3. Lupeol Prevents 7-Keto-Cholesterol-Induced Lipid Accumulation and Enhances Reactive Oxygen Species (ROS) Production in *M*_(IFN-*γ*/LPS)_ Macrophages

**Figure 4:**
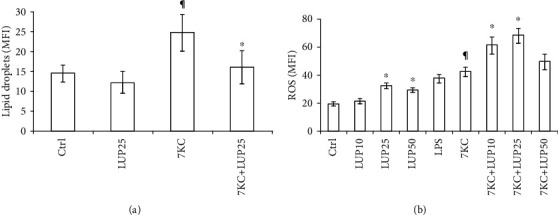
Effect of lupeol on intracellular lipid levels and reactive oxygen species (ROS) production. *M*_(IFN-*γ*/LPS)_ macrophages pretreated with lupeol for 1 hour were stimulated with 7-keto-cholesterol (7KC) for 20 hours. THP-1 *M*_(IFN-*γ*/LPS)_ macrophages were analyzed for lipid droplets (a) and primary *M*_(IFN-*γ*/LPS)_ macrophages were analyzed for ROS generation (b) by flow cytometry. Results are expressed as mean ± SD from three independent experiments. ^∗^Untreated vs. lupeol; ^¶^Untreated control vs. 7KC-treated group; °7KC-treated group vs. 7KC+lupeol treated groups.

To further confirm the ability of lupeol to counteract the atherosclerotic process, we used the 7KC to increase lipid accumulation in *M*_(IFN-*γ*/LPS)_ polarized THP-1 macrophages. By flow cytometric analysis, we studied the ability of lupeol to reduce the accumulation of the LipidSpot™ dye induced by 7KC on THP-1 *M*_(IFN-*γ*/LPS)_ macrophages. As expected, the mean fluorescence intensity for the LipidSpot™ significantly increased after the 20-hour exposure of *M*_(IFN-*γ*/LPS)_ macrophages to 7KC (in Figure 4(a)). Of note, the 25 *μ*M lupeol prevented 7KC-induced lipid accumulation in *M*_(IFN-*γ*/LPS)_ macrophages (in Figure 4(a)). Previous studies showed that inflammatory macrophages release ROS, thus exacerbating oxidative stress in atherosclerosis [30, 31]. For this reason, we analyzed flow cytometry ROS production in primary *M*_(IFN-*γ*/LPS)_ macrophages stimulated with 7KC after pretreatment or not with lupeol. As expected, we observed that the treatment of the *M*_(IFN-*γ*/LPS)_ macrophages with 7KC (15 *μ*M) induced a significant increase in ROS production (*P* < 0.001, in Figure 4(b)). Of note, lupeol pretreatment resulted in an additive effect in ROS production in macrophages stimulated with 7KC. The maximal effect of lupeol was observed at a concentration of 25 *μ*M (*P* < 0.001; in Figure 4(b)).

### 3.4. Lupeol Counteracts Dysregulated Autophagy Induced by 7-Keto-Cholesterol in *M*_(IFN-*γ*/LPS)_ Macrophages

**Figure 5:**
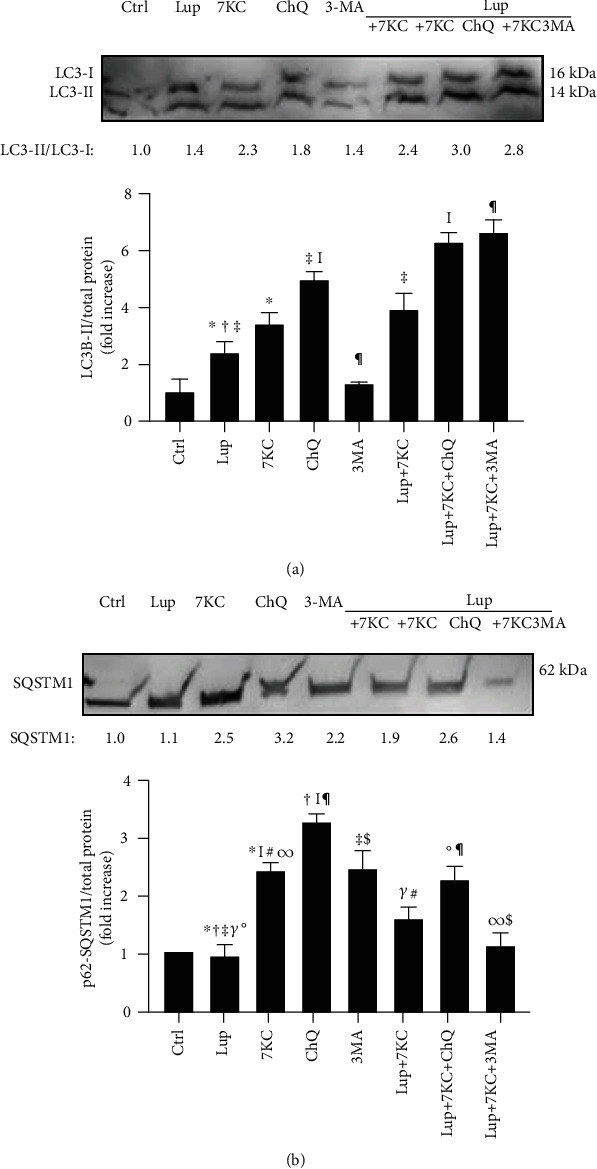
Western blotting analysis of autophagy markers in *M*_(IFN-*γ*/LPS)_ macrophages. Lupeol enhances autophagy on *M*_(IFN-*γ*/LPS)_ macrophages and counteracts dysregulated autophagy induced by 7-Keto-cholesterol (7KC). *M*_(IFN-*γ*/LPS)_-polarized THP-1 macrophages were stimulated or not with 25 *μ*M lupeol for 1 hour and further stimulated with 7KC for 20 hours in the presence of the autophagy inhibitors ChQ or 3MA. Western blotting analysis of (a) LC3-I/II and (b) P62-SQSTM1 were performed in the whole-cell lysates of macrophages. Data are expressed relative to the control (fold increase) as mean ± SD of 4 independent experiments. Values mentioned are the ratio of LC-3-II to LC3-I. Symbols indicate significant differences tested by one-way ANOVA.

A derivative molecule of lupeol has been shown to induce cell death in cancer cells by inducing autophagy rather than apoptosis and necrosis by accumulating ROS [21]. Adhering to this evidence, we next measured the effects of lupeol on the modulation of autophagy dysfunction in *M*_(IFN-*γ*/LPS)_ macrophages induced by 7KC [29]. By using western blotting analysis, we evaluated the expression of the autophagic marker LC3-I/II, a widely used marker to monitor the autophagic process and the marker for autophagic clearance p62/sequestosome 1 (SQSTM1) on *M*_(IFN-*γ*/LPS)_-polarized THP-1 macrophages and on *M*_(IFN-*γ*/LPS)_-polarized primary macrophages pretreated with lupeol and further exposed or not to 7KC. In preliminary experiments, *M*_(IFN-*γ*/LPS)_ macrophages derived from THP-1 and primary macrophages showed similar changes in the expression of autophagy markers when compared with respective unstimulated samples; in fact, both cells upregulated autophagy markers in response to 7KC (see Figure S2 in the Supplementary Material for comprehensive result analysis). As shown in Figure 5(a), the addition of lupeol or 7KC induced an increase in the transient autophagosomal membrane-bound form of LC3 (LC3-II) in *M*_(IFN-*γ*/LPS)_ macrophages. It is already known that LC3-II could accumulate due to enhanced autophagosome formation or impaired autolysosomal degradation [32]. To rule out the possibility that the increase of LC3-II was due to inhibited autolysosomal degradation, rather than autophagy stimulation and the respective autophagosome formation, we further evaluated LC3-II flux. For this purpose, *M*_(IFN-*γ*/LPS)_-polarized THP-1 macrophages were incubated with lupeol and 7KC in the presence of the lysosomal protein degradation inhibitor ChQ or of the autophagy inhibitor 3-MA. In these conditions, there was an increase in LC3-II induced by lupeol and 7KC in the presence of ChQ or 3MA, and this increase was significantly higher than in cells treated with inhibitor alone. LC3 has been proposed to function as a receptor for p62/SQSTM1. The LC3-p62 complex is preferentially degraded by autophagy and markedly accumulates in autophagy-deficient cells [33]. Since p62 accumulates when autophagy is inhibited and decreases when autophagy is induced, therefore, p62-SQSTM1 could be used as a marker to study autophagic flux [33]. In our experiments, lupeol inhibited the p62 protein levels, whereas an increase in the p62 protein levels was observed in 7KC, ChQ, and 3-MA treated cells (in Figure 5(b)), which suggests that p62/SQSTM1 autophagic degradation was inhibited by 7KC, similarly to ChQ and 3-MA, whereas lupeol increased the autophagic flux in macrophages. Of note, lupeol was able to significantly reduce the p62 accumulation in *M*_(IFN-*γ*/LPS)_-polarized THP-1 macrophages when these cells were treated with 7KC in the presence or absence of autophagy inhibitors (in Figure 5(b)).

## 4. Discussion

The identification of the pathological role played by polarized macrophages has resulted in an increased focus on this paradigm for the identification of new therapeutic approaches [16, 27]. Lupeol is a ubiquitously distributed pentacyclic triterpene of the edible vegetables, fruits, and many medicinal herbs [17]. Lupeol plays an anti-inflammatory role in several inflammatory disease models such as carrageenan-induced inflammation [34], A23187-stimulated macrophages [35], a mouse model of arthritis [36], allergic airway inflammation [19], and LPS-treated macrophages [35].

In our previous study, we demonstrated that 7KC polarizes macrophages toward a proinflammatory state [9]; therefore, it is worthwhile to use this *in vitro* model to investigate the ability of a compound to switch human macrophages from a M1 proinflammatory phenotype (high IL-12 and IL-1*β* production, high HLA-DR expression, and low endocytosis ability) to a M2 anti-inflammatory phenotype (high IL-10 and TGF-*β* production, high CD36 expression, and high endocytosis ability) [16, 27]. Thus, we have considered lupeol to meet the aims of our study.

This study demonstrating an anti-inflammatory and immunomodulatory activity of lupeol in human *M*_(IFN-*γ*/LPS)_ macrophages challenged with the inflammatory cholesterol oxidation product 7KC indicates lupeol as a promising therapeutic agent for atherosclerotic disease.

We first determined the effects of lupeol on the release of proinflammatory and anti-inflammatory cytokines in the *M*_(IFN-*γ*/LPS)_ macrophages and in 7KC-treated-*M*_(IFN-*γ*/LPS)_ macrophages. Our study demonstrated the ability of lupeol to regulate macrophage polarization by reducing the release of the proinflammatory cytokine IL-12 and by increasing the release of the anti-inflammatory cytokines IL-10 and TGF-*β*, thus driving cells toward a M2 anti-inflammatory phenotype. Lupeol was also able to counteract the proinflammatory signalling triggered in macrophages by 7KC represented by the downregulation of IL-12 and IL-1*β* production and upregulation of IL-10. Our results agree with a previous report showing that lupeol significantly inhibited proinflammatory cytokine production in macrophages and suppressed LPS-induced NF-*κ*B activity in inflammatory bowel disease [37]. While no cytotoxicity was observed upon lupeol treatment at the tested concentrations, the lack of cytokine production increase observed at the highest lupeol concentration suggests a possible interference of lupeol with immune cell regulation mechanisms as apoptosis pathway [38]. Furthermore, another natural compound, luteolin, inhibits inflammation by regulating the polarized phenotypes of macrophages and downregulates the release of proinflammatory cytokines [13].

Surface marker phenotyping confirmed that 7KC-treated-*M*_(IFN-*γ*/LPS)_ macrophages showed an increased expression of the M1 activation marker HLA-DR [9], suggesting an upregulation of macrophage function as antigen-presenting cells that favor the activation of adaptive immune responses. In our experiments on primary macrophages, after 20 hours of exposure, 7KC did not alter the expression of the macrophage class B scavenger receptor CD36, a member of the scavenger receptor family involved in M2 polarization [39]. Hayden et al. [31] demonstrated that macrophages generated by 7KC treatment of THP-1 cells for 7 days increased CD36 expression about by 2-fold. A reason for this discrepancy between the effects of 7KC on primary macrophages and THP-1-macrophages may be due to the different intracellular lipid levels reached by the cells, which are the expression of both different culture conditions and cell differentiation state [40, 41]. The upregulation of CD36 observed in the *M*_(IFN-*γ*/LPS)_ macrophages to lupeol is likely to positively influence the lipid uptake into cells, further increasing their anti-inflammatory clearance activity.

A further evidence of lupeol anti-inflammatory effects on *M*_(IFN-*γ*/LPS)_ macrophages is its ability to increase the endocytic capacity of these cells. Similar results were observed in previous experiments with macrophage polarization in colorectal cancer cells [42].

Previous studies showed that excess-free cholesterol is stored as lipid droplets in macrophages and produces foam cell morphology [31, 43]. Since foam cell formation due to lipid droplets accumulation in macrophages is believed to play a crucial role in the progression of early atherosclerotic lesions and subsequent inflammation [44], we next evaluated the effects of lupeol pretreatment on 7KC-induced lipid droplets accumulation in *M*_(IFN-*γ*/LPS)_ macrophages. The mean fluorescence intensity profiles reveal that lupeol reduces the accumulation of lipid droplets in macrophages treated with 7KC, thus further suggesting that the exposure of *M*_(IFN-*γ*/LPS)_ macrophages with lupeol influences the polarization of proinflammatory macrophages toward a less proinflammatory phenotype through the influence on the lipid metabolism, similarly to what was observed in response to several other compounds modulating foam cell behaviour and inhibiting lipid accumulation [45]. Our results are in line with other studies on triterpenoids that have been shown to inhibit the accumulation of lipid droplets in macrophages [43]. Lupeol has been found to prevent the hypertrophic cardiac histopathology, the lipid abnormalities, and pathological biochemical changes induced by hypercholesterolemia [46]. Overall, available data on the potential benefit of lupeol as a natural lipid-lowering compound appear promising, thus further research on its beneficial effects needs to be performed.

Another crucial parameter in the pathogenesis of atherosclerosis is the ROS secretion. Our results on the increased ROS secretion due to 7KC stimulation are in agreement with previous studies showing the secretion of ROS by inflammatory macrophages that exacerbate oxidative stress in atherosclerosis [28, 30]. The finding that lupeol pretreatment significantly increased the ROS generation in our study is in line with the observation demonstrating that a derivative molecule of lupeol is able to induce the accumulation of ROS [20]. Notably, mitochondria and ROS are essential for autophagy stimulation [47–49].

Accumulating evidence shows that the dysfunctional autophagy plays a key role in atherosclerosis [24–26]. The impaired macrophage autophagy increases the immune response in obese mice by promoting proinflammatory M1 macrophage polarization [50]. Of note, a derivative molecule of lupeol induces cell death in a cancer cell line by inducing autophagy rather than apoptosis and necrosis [21]. Recently, a study showed that autophagy induction reduces 7KC-mediated cell death and reduces cellular lipid accumulation [29]. Our results suggest that lupeol is able to induce both autophagosome formation, as indicated by increased LC3 levels, and increased autophagy lysosomal degradation pathway, as marked by the lack of p62 accumulation [49].

Further studies on the characterization of ROS production induced by lupeol, over more extended time intervals, will establish whether this production simply regulates the autophagic pathway without leading to the collapse of the potential of the mitochondrial membrane or it can progress to autophagic cell death.

Taken together, our data provide evidence that lupeol has a cytoprotective effect toward 7-keto-cholesterol-induced proinflammatory signalling by efficiently switching the proinflammatory phenotype toward an anti-inflammatory one, which may be due to the activation of the autophagy pathway by increasing ROS production, to the modulation of cellular lipid accumulation and to an overall reduction of proinflammatory cytokines. Similar results have also been observed with urolithin A that shows high anti-inflammatory potential by inhibiting M1 polarization in macrophages and increasing the autophagic flux [51].

Extensive research so far on efficacy, safety, and pharmacokinetics profile of lupeol has shown that the oral administration of lupeol (<200 mg/kg) does not cause any systemic toxicity in animal models [52–54]. In a randomized controlled clinical trial (NCT02152865), the administration of lupeol was found to be safe and nontoxic for the treatment of oral malignant melanoma [55]. These findings suggest that lupeol should be studied further as anti-inflammatory therapeutics for future applications in humans. Lupeol has been shown to inhibit NF-*κ*B and to increase FGF-2, TGF-*β*1, and collagen III levels, followed by the downregulation of IL-6 and subsequent upregulation of IL-10 levels in a wound healing model in diabetic patients [56]. Since our data confirm the anti-inflammatory activity of the lupeol against 7KC in *M*_(IFN-*γ*/LPS)_ macrophages by suppressing inflammasome and activating autophagy, they suggest the possible usefulness of this molecule in the assessment of new potential therapeutic strategies for plaque regression.

## 5. Conclusions

Our results strengthen previous findings on the immunomodulatory effects of lupeol on innate immune cells and depict the usefulness of lupeol as an adjunctive drug to counteract the proatherogenic oxysterol signalling within the atherosclerotic plaque through the activation of autophagy and inhibition of proinflammatory cytokines.

## Data Availability

The data used to support the findings of this study are included within the article.

## Supplementary Materials

Supplementary Materials Supplementary Figure 1: the effect of lupeol on cell vitality (measured by Trypan blue exclusion assay) and cell morphology (light microscope imaging) of 7-keto-cholesterol-treated *M*_(IFN-*γ*/LPS)_ macrophages. Supplementary Figure 2: Western blotting analysis of LC3-I/II and p62-SQSTM1 in the whole-cell lysates of primary *M*_(IFN-*γ*/LPS)_ macrophages and THP-1 *M*_(IFN-*γ*/LPS)_ macrophages in response to 7-keto-cholesterol (a) or in response to lupeol plus 7KC (b).
