# Supplementary material for: Lupeol Counteracts the Proinflammatory Signalling Triggered in Macrophages by 7-Keto-Cholesterol: New Perspectives in the Therapy of Atherosclerosis
Source: Oxid Med Cell Longev. 2020 Sep 27;2020:1232816. doi: 10.1155/2020/1232816 (PMC7537694; doi:10.1155/2020/1232816)
Supplement: Supplementary Materials — Supplementary Figure 1: the effect of lupeol on cell vitality (measured by Trypan blue exclusion assay) and cell morphology (light microscope imaging) of 7-keto-cholesterol-treated M(IFN-γ/LPS) macrophages. Supplementary Figure 2: Western blotting analysis of LC3-I/II and p62-SQSTM1 in the whole-cell lysates of primary M(IFN-γ/LPS) macrophages and THP-1 M(IFN-γ/LPS) macrophages in response to 7-keto-cholesterol (a) or in response to lupeol plus 7KC (b). [file 1232816.f1.docx]

Supplementary materials to

**Lupeol counteracts the pro-inflammatory signalling triggered in macrophages by**

**7-cheto-cholesterol: new perspectives in the therapy of atherosclerosis**

by Sarmistha Saha et al.,

***i***

***ii***


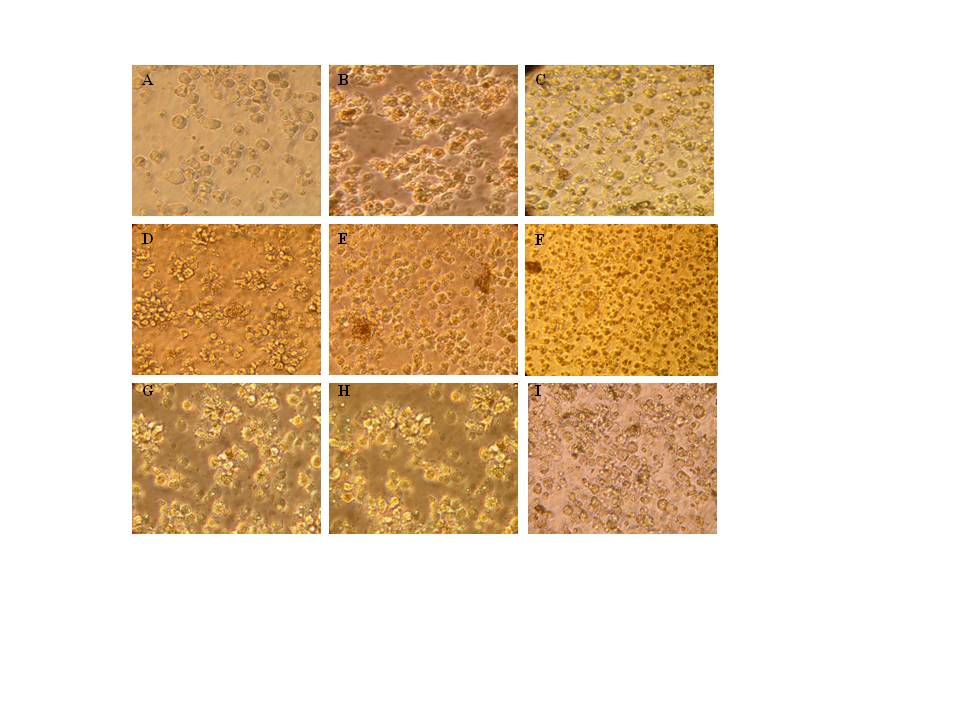


**Figure S1.** *The effect of lupeol on cell vitality and cell morphology of 7-keto-cholesterol-treated M_(IFN+LPS)_ macrophages.* Primary M_(IFN+LPS)_ macrophages were seeded in a 96-well plate and treated with different concentrations of lupeol and further stimulated or not with 7-keto-cholesterol (7KC) for 20 hours at 37 °C. ***i***  Cell viability measured by Trypan blue exclusion assay. Dose-response experiments demonstrated that 50 µM was the highest tolerated concentration of lupeol that didn’t affect M_(IFN+LPS)_-macrophage viability. Results are expressed as mean value ± SD of 3 independent experiments. P values were tested by one-way ANOVA. *Untreated *vs* lupeol 100 µM: p < 0.05. ***ii*** Light microscope imaging. Light micrograph with magnification 100 x of (A) untreated control cells, (B) 7-keto-cholesterol, (C) LPS, (D) lupeol 10 µM, (E) lupeol 25 µM, (F) lupeol 50 µM, (G) 7KC + lupeol 10 µM, (H) 7KC + lupeol 25 µM, (I) 7KC + lupeol 50 µM. A normal and distinct feature of macrophage morphology was observed in response to lupeol (10-50 µM) and 7KC (15 µM) or to a combination of the two compounds. LPS (200 ng/mL) was used as positive control of macrophage activation. The increased cytoplasmic ratio, pseudopodia and vacuolar system were observed in all samples, thus indicating a high phagocytic activity typical of macrophage cells. Results shown are representative images of 3 independent experiments.


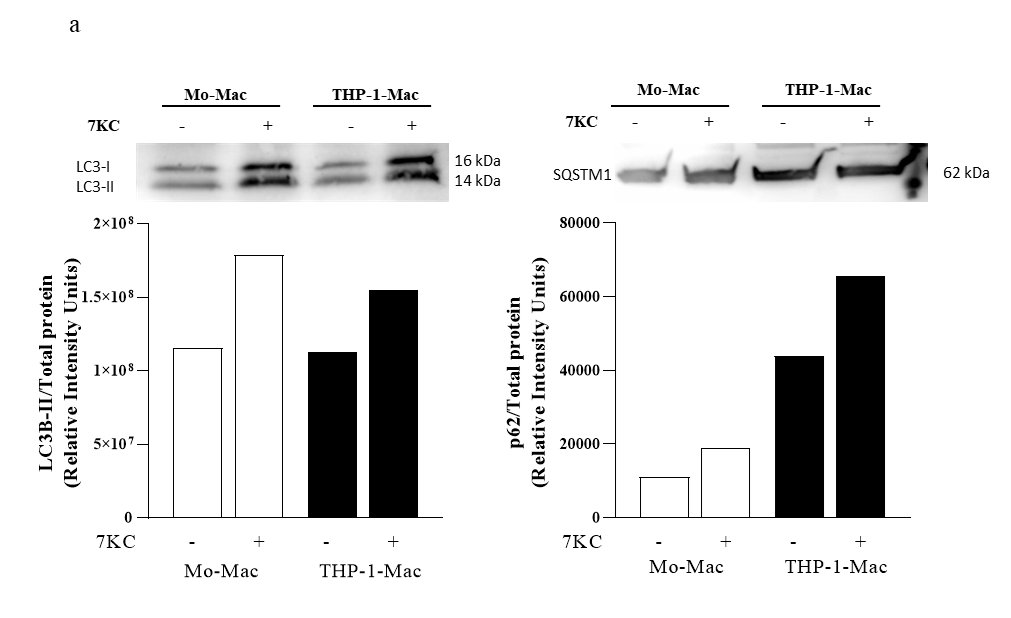


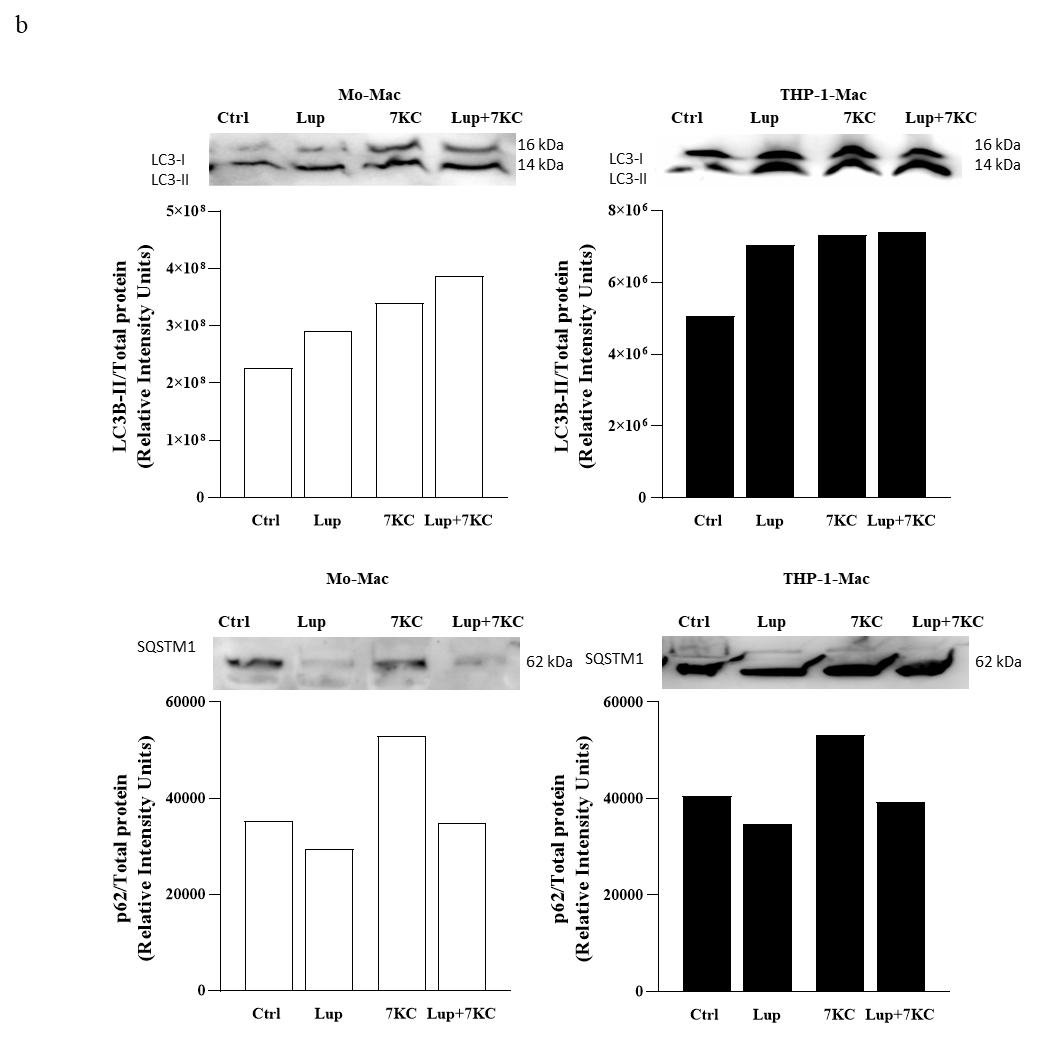


**Figure S2.** *7-Keto-cholesterol (7KC) induced a dysregulated autophagy both on primary M_(IFN+LPS)_ macrophages and on THP-1 M_(IFN+LPS)_ macrophages and this effect was counteracted by lupeol pretreatment*.

Western blotting analysis of LC3-I/II and p62-SQSTM1 performed in the whole-cell lysates of macrophages. Protein immunoreactive bands were visualized by chemifluorescence with the Clarity Western ECL Substrate (Bio-Rad) in a ChemiDoc Imaging System (Bio-Rad). The optical density of the bands was quantified with the Imagelab 6.0.1 (Bio-Rad). The results were normalized to total protein and expressed as relative intensity units. (a) Human primary M_(IFN-γ/LPS)_ polarized macrophages (monocyte-derived macrophages: Mo-Mac) and M_(IFN+LPS)_-polarized THP-1 macrophages (THP-1-Mac) were stimulated with 7-Keto-cholesterol (7KC) for 20 hours. In response to 7KC both macrophage models showed similar changes in the expression of autophagy markers, in fact both cells up-regulated the expression levels of LC3-I/II and p62 when compared with respective unstimulated samples. Representative Western blot images and relative densitometric bar graphs are reported (N = 2) (b) Human primary M_(IFN-γ/LPS)_ polarized macrophages (Mo-Mac) and M_(IFN+LPS)_-polarized THP-1 macrophages (THP-1-Mac) were pre-treated with 25 µM lupeol and further stimulated with 7KC for 20 hours. Lupeol counteracted dysregulated autophagy induced by 7KC in both primary macrophages and THP-1 macrophages. In both cell models, lupeol increased LC3 levels and prevented the p62 accumulation. Western blot images and relative densitometric bar graphs are reported.
